# Non-Linear Measures of Postural Control in Response to Painful and Non-Painful Visual Stimuli

**DOI:** 10.3390/e25111561

**Published:** 2023-11-19

**Authors:** Alexandre Vonesch, Cassandre Duhot, Thierry Lelard, Guillaume Léonard, Michalina Błażkiewicz, Harold Mouras

**Affiliations:** 1 UR-UPJV 4559 LNFP Laboratoire de Neurosciences Fonctionnelles et Pathologies, Université de Picardie Jules Verne, 80054 Amiens, France; 2 UR UPJV 3300 APERE Adaptation Physiologiques à l’Exercice et Réadaptation à l’Effort, Université de Picardie Jules Verne, 80054 Amiens, France; thierry.lelard@u-picardie.fr; 3 Research Centre on Aging, CIUSSS de l’Estrie—CHUS, Sherbrooke, QC J1H 4C4, Canada; guillaume.leonard2@usherbrooke.ca; 4 School of Rehabilitation, Faculty of Medicine and Health Sciences, Université de Sherbrooke, Sherbrooke, QC J1H 5N4, Canada; 5 Faculty of Rehabilitation, The Józef Piłsudski University of Physical Education in Warsaw, 00-809 Warsaw, Poland; michalina.blazkiewicz@awf.edu.pl

**Keywords:** posturography, non-linear measures, painful stimuli perception

## Abstract

Over the past decade, researchers have focused on studying the functional context of perceiving painful stimuli, particularly concerning the posturographic correlates of emotional processing. The aim of this study was to investigate the differential modulation of non-linear measures characterizing postural control in the context of perceiving painful stimuli. The study involved 36 healthy young participants who, while standing, viewed images depicting feet and hands in painful or non-painful situations, both actively (by imagining themselves affected by the situation) and passively. For Center of Pressure (COP) displacement, three non-linear measures (Sample Entropy, Fractal Dimension, and Lyapunov exponent) were calculated. The results suggest lower values of FD and LyE in response to active stimulation compared to those recorded for passive stimulation. Above all, our results pledge for the usefulness of the Lyapunov exponent for assessing postural modulation dynamics in response to painful stimuli perception. The feasibility of this calculation could provide an interesting insight in the collection of biomarkers related to postural correlates of emotional processes and their modulation in neurological disease where socio-affective functions can be often impaired before cognitive ones.

## 1. Introduction

Over the last decade, investigation of the interaction between emotion and motor skills has gained new impetus through research methods applied in different disciplines, from sports science to socio-affective neuroscience. Posturography is a technique used to assess postural control under static or dynamic conditions by measuring displacements of the body’s Center of Pressure (COP) in the anteroposterior (AP) and mediolateral (ML) directions [1,2]. In particular, this technique is interesting for objectively assessing approach–avoidance behavior toward a stimulus [3,4]. The links between postural control and emotion have been studied in the functional model of pain perception and empathy for pain. These investigations have successively demonstrated (i) a differential postural response to the presentation of painful versus non-painful stimuli [5]; (ii) a modulation of the postural response by the instruction given to the participant to “imagine oneself in painful situations” [6]; and (iii) a modulation of the postural response by the level of pain [7]. In the first case, authors have used so-called classical methods of analysis, focusing mainly on the COP path length and COP velocity, sometimes considered separately in the AP and ML directions. Regarding the functional context of painful stimuli perception, it should be emphasized that previous studies highlighted the importance of the temporal modulation of the postural correlates of painful stimuli perception [6] (for example, demonstrating differential postural approach–avoidance tendencies along the time of painful stimulus presentation). However, to date, this role has only been explored through the lens of non-statistical, descriptive approaches such as the extraction of the time course of posturographic variables. While the results obtained are interesting, they do not provide a detailed understanding of postural control dynamics and do not provide any information regarding their temporality.

Given that the time series of COP in both the anteroposterior and mediolateral directions represent the output of a unique, complex, and integrated system, it appears necessary to study its regularity, stability, and adaptability. Non-linear measures provide such opportunities [8,9]. They are able to capture the temporal component of COP displacement variability in terms of how motor behavior develops over time [10]. These measures include, among others, Sample Entropy (SampEn), which informs on the regularity of postural sway; Fractal Dimension (FD), which indicates the complexity of the COP signal by describing its shape; and Lyapunov exponent (LyE), which characterizes the chaotic behavior of the signal [10]. Higher SampEn values indicate more irregular postural sway patterns, suggesting less stable postural control. Lower values, on the other hand, suggest more predictable and regular postural sway, indicating better stability and control. FD is another mathematical measure used to characterize the complexity or self-similarity of geometric patterns [11]. In the context of postural control, FD refers to the scaling properties of postural sway, providing a quantitative measure of the degree of self-similarity or complexity within these patterns. Studies have suggested that a higher FD in postural sway patterns is associated with better postural control and balance performance. Results from these studies indicate that individuals with more complex and self-similar postural sway patterns tend to exhibit more adaptive and robust postural control strategies. Conversely, a lower FD may point to reduced complexity and less optimal postural control. As previously mentioned, postural sway can exhibit complex and non-linear dynamics. The Lyapunov exponent gives a gauge of chaotic behavior in these dynamics. A positive LyE indicates sensitive dependence on initial conditions and suggests chaotic behavior in postural sway. It implies that small differences in initial body position or perturbations can lead to significantly different postural sway patterns over time. On the other hand, a negative or zero LyE suggests more stable and predictable postural control, with trajectories converging or staying close together [10].

To date, these measures have been sparingly used to study the posturographic correlates of emotions. Błażkiewicz et al. [12], while assessing the relationships between postural control, as evaluated using non-linear measures, and the five-factor model of personality, questioned whether personality traits can determine postural control in healthy young adults. Their findings indicated that factors such as visual control and the size of the support area, rather than personality traits, played a significant role in describing postural control. Stins et al. [13] reported lower SampEn in a height condition and interpreted it as an increase in attentional resources focused on postural control in an affective setting. More recently, Fischer et al. [14] reported an increase in SampEn in response to postural threat conditions, which was interpreted as a shift towards more automatic postural control.

To our knowledge, no study has used these parameters to explore the neural and postural correlates in the perception of pain. As explained above, the analysis of the temporal modulation of the postural correlates of painful stimuli perception was, to date, highly exploratory. Thus, based on the recent literature on the importance of non-linear measures for the exploration of posture modulation, we hypothesized a differential modulation of non-linear measures of COP’s displacement (i) in response to painful stimuli as compared to non-painful ones and (ii) in the active stimulation condition as compared to the passive one.

## 2. Materials and Methods

### 2.1. Participants

The study included 36 healthy young participants (16 men and 20 women; mean age: 24.2 ± 6.1 years old). The number of participants included in this research was similar (and even more important) to the samples included in our preceding studies focusing on the postural correlates of painful stimuli perception. Exclusion criteria were as follows: (i) known visual, ocular, or motor disorders; (ii) the use of medications that could disrupt motor and psychological activity, including sleep treatments; (iii) a history of photosensitive epilepsy; (iv) individuals with a history of migraines; (v) those with known static or dynamic balance abnormalities; (vi) pregnant women; (vii) individuals reporting significant motion sickness requiring medication. Several conditions were monitored before and after the experiment: (i) levels of anxiety (assessed using the State-Trait Anxiety Inventory, STAI) [15,16]; (ii) fatigue (measured using the Pichot Fatigue Scale) [17]; (iii) sleepiness (evaluated with the Epworth Sleepiness Scale) [18]. Participants provided informed consent and received an information letter before beginning the experiment. The study received approval from the CER of Université Paris Saclay (Orsay, France), and all experimental procedures adhered to the ethical standards outlined in the Declaration of Helsinki.

### 2.2. Stimuli and Procedure

The study used 20 pictures depicting feet and hands in painful or non-painful situations, randomly selected from a database validated by previous studies [19]. The presentation of these stimuli was controlled by a computer running the E-Prime software (Version 2, Psychology Software Tools, Inc., Pittsburgh, PA, USA).

Participants stood barefoot in the middle of the force plate (AMTI, Watertown, MA, USA), feet inside the marking. Footprints were marked on the force platform to ensure the maximum reproducibility of participants’ foot positions from one trial to another one. From one trial to the next, the participant was asked to keep the same position on the force platform, i.e., they were asked to maintain a comfortable stance with their arms alongside their body and to stay still. The stimuli were displayed on a screen 1.20 m in front of the participant. During the experiment, the room door was closed, the lights were turned off, and the windows were covered. The researcher was outside the room and was able to follow the stimuli on the screen. Stimuli were presented under two experimental conditions corresponding to two runs: (1) passive stimulation, where participants were asked to “look at the pictures”; (2) active stimulation, where participants were asked to “look at the pictures while imagining you are the person living these situations”. Each run was made of 5 non-painful and 5 painful pictures, all different, presented randomly for 20 s and separated by a gray screen for 6 s, followed by a screen of 2 s with the instruction “Stay as still as possible” and the instruction “look at the picture” for the passive condition or the instruction “imagine that you are the person living through this situation” for the active condition. A screen displaying a fixation crosshair was then presented for 2 s. The inter-stimulus period was used to ensure that the posture measured for the subsequent image did not interfere with the return to the starting position after the presentation of the last stimulus. The stimuli presented in each condition varied for each participant, and a gray screen background was used during the inter-stimulus period (gray screen, instructions, fixation cross) to prevent a potential physiological flash response triggered by the presented image. Following the presentation of the five stimuli in each session, the participants had a 2-min break, during which they were asked to sit in a chair placed behind.

### 2.3. Measures

For each experimental condition, posturographic data were recorded at a sample rate of 100 Hz using the AMTI NetForce software (https://www.amti.biz/product/netforce/) and a AMTI force plate (AMTI, Watertown, MA, USA) constituted of four force gauges measuring postural reaction forces and moments exerted by the feet.

The calculation of COP coordinates was carried out using the equations provided within the AMTI Biomechanics Force Platform Installation Manual (version 4.4, April 2017), $COPx=\frac{-My}{Fz}$ and $COPy=\frac{Mx}{Fz}$, where Mx and My are the output moments and Fz is the z force component of the resultant applied force.

The time series of COPx (ML direction) and COPy (AP direction) measurements corresponding to 20 s of presentation for each image were combined into a single time series separately for the AP and ML directions for each experimental condition (passive–painful, active–painful, passive–painful, active–painful).

### 2.4. Non-Linear Mesaures

The study employed 3 non-linear measures to assess COP dynamics: SampEn, LyE, and FD. These non-linear coefficients were computed separately for the AP and ML directions using MatLab software (V2021a, MathWorks, Natick, MA, USA).

SampEn calculates the probability that a sequence of N-data points, having repeated itself within a tolerance r for m points, will also repeat itself for m+1 points without allowing self-matches: $SampEn\left(m,r,N\right)=-ln\left(\frac{A^{m}\left(r\right)}{B^{m}\left(r\right)}\right)$. B represents the total number of matches of length m, while A represents the subset of B that also matches for m+1. Thus, a low SampEn value arises from a high probability of repeated template sequence in the data, hence greater regularity. For calculating the SampEn, we used the MatLab codes obtained from the Physionet tool [12] with “default” parameters: m = 2, r = 0.2 × SD, where SD is the standard deviation of the COP time series.

LyE assesses a system’s local stability, reflecting its resistance to small internal perturbations such as the natural fluctuations that occur while maintaining an upright stance. The idea of using LyE to identify chaos in a system comes from the assumption that if the average distance between 2 points grows exponentially, the system is sensitive to changes in initial conditions, so the LyE is higher than zero: $d\left(t\right)={Ce}^{LyEt}$, where d(t) represents the average divergence at time t, and C is a constant that normalizes the initial separation. Accordingly, the existence of a positive LyE is commonly taken as a necessary and sufficient condition for chaos in the system.

FD assesses the complexity of the COP signal by examining its shape, with changes that may suggest the modulation of postural control strategy. A low FD value indicates a highly stationary signal over time, while a high FD indicates a non-stationary signal [10]. In this study, the Higuchi algorithm [20] was used to calculate FD.

### 2.5. Statistical Analysis

The analysis was performed using the JASP software (V 0.18.1, JASP Team, Amsterdam, The Netherlands). Statistical significance was set at *p* < 0.05. Two-way repeated-measures ANOVAs with Bonferroni post hoc tests were used to determine if there was a statistically significant interaction effect between stimulation (active or passive) and valence (painful or non-painful stimuli). Statistical tests were conducted independently for all 3 measures (SampEn, LyE, and FD) and separately for COP displacement in the AP and ML directions.

The same analyses were then performed to look at the effect of direction on the behavior of non-linear parameters, looking for the presence of interactions between the stimulation factors, valence, and direction.

## 3. Results

### 3.1. COP Time Series Analysis for Anteroposterior Direction

**Figure 1:**
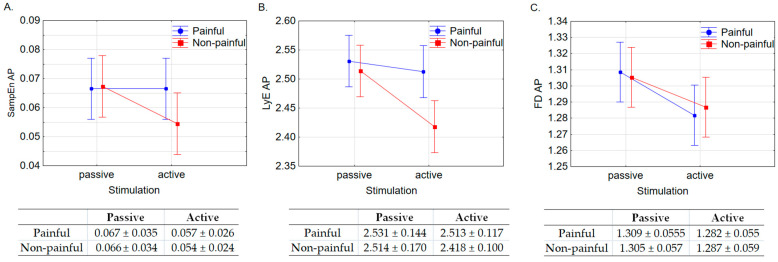
ANOVA results, mean and standard deviations for the stimulation (passive, active) and valence (painful, non-painful) factors for (**A**). Sample Entropy (SampEn), (**B**). the Lyapunov exponent (LyE) and (**C**). Fractal Dimension (FD) for the time series COP in anteroposterior (AP) direction.

The main results of the ANOVA for the anteroposterior direction are presented in Figure 1.

For SampEn (Figure 1A), a non-significant effect of the valence factor (F(1, 35) = 0.118, *p* = 0.733), the stimulation factor (F(1, 35) = 3.079, *p* = 0.088), as well as a non-significant interaction effect between valence and stimulation (F(1, 35) = 0.682, *p* = 0.414) were obtained.

For LyE (Figure 1B), significant effects for both the valence (F(1, 35) = 9.937, *p* = 0.003) and stimulation factors (F(1, 35) = 6.145, *p* = 0.018), along with a significant interaction effect between these factors (F(1, 35) = 8.878, *p* = 0.005), were noted. LyE values were significantly higher, albeit by a small margin of 2.26%, during observations of painful pictures compared to those without pain. Likewise, LyE values were significantly higher, with a difference of 2.31%, during passive stimulation compared to active stimulation. Post hoc analyses looking at the interaction between valence and stimulation revealed that LyE values were significantly lower during active observations of images without pain compared to those with pain (*p* = 0.001) and without pain (*p* = 0.003) under passive conditions. LyE values were also significantly lower (*p* < 0.001) during active observations of images without pain compared to passive observations of images with pain.

For FD (Figure 1C), the valence factor yielded a non-significant effect (F(1, 35) = 0.095, *p* = 0.760), while the stimulation factor produced a significant effect (F(1, 35) = 19.106, *p* < 0.001), with FD values being significantly higher for passive conditions. There was no significant interaction effect between these factors (F(1, 35) = 2.507, *p* = 0.122).

### 3.2. COP Time Series Analysis for Mediolateral Direction

**Figure 2:**
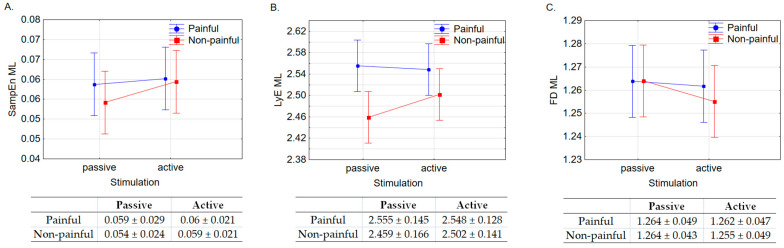
ANOVA results, mean and standard deviations for the stimulation (passive, active) and valence (painful, non-painful) factors for (**A**). Sample Entropy (SampEn), (**B**). the Lyapunov exponent (LyE) and (**C**). Fractal Dimension (FD) for the time series COP in the mediolateral (ML) direction.

Results from the ANOVA showed a different pattern of results for the mediolateral direction and the anteroposterior direction, as shown in Figure 2.

For SampEn (Figure 2A), no significant effect was observed for either the valence factor (F(1, 35) = 1.278, *p* = 0.266) or the stimulation factor (F(1, 35) = 0.54, *p* = 0.467). The interaction between stimulation and valence was not significant (F(1, 35) = 1.521, *p* = 0.226).

In the case of LyE (Figure 2B), a significant effect was observed for valence (F(1, 35) = 14.945, *p* < 0.001). Significantly higher LyE values were recorded for observations of images depicting pain compared to those without pain. A non-significant effect was noted for the stimulation factor (F(1, 35) = 0.497, *p* = 0.485). Moreover, the interaction effect between these factors was not significant (F(1, 35) = 2.100, *p* = 0.156).

For FD (Figure 2C), a non-significant effect was detected for both the valence (F(1, 35) = 1.170, *p* = 0.287) and stimulation (F(1, 35) = 1.830, *p* = 0.185) factors. Likewise, the interaction between these factors was non-significant (F(1, 35) = 0.885, *p* = 0.353).

### 3.3. Effect of Direction on the Behavior of Non-Linear Parameters

The 2 (stimulation: passive, active) X 2 (valence: painful, non-painful) X 2 (direction: AP, ML) ANOVA revealed no significant effect for SampEn for the 3 factors and their combinations. For FD, a significant effect of stimulation (F(1, 35) = 15.974, *p* < 0.001) and direction (F(1, 35) = 24.978, *p* < 0.001) was observed. Bonferroni post hoc tests revealed that FD values in passive conditions significantly exceeded those in active conditions (*p* < 0.001), and FD values for the AP direction significantly exceeded those for the ML direction (*p* < 0.001). Additionally, a significant interaction effect of stimulation and direction on FD values was found (F(1, 35) = 7.946, *p* = 0.008) (Table 1).

A valence effect was observed for LyE (F(1, 35) = 27.666, *p* < 0.001), with significantly higher values when participants were viewing painful images compared to non-painful images (*p* < 0.001). There was also a significant interaction between stimulation and direction (F(1, 35) = 4.300, *p* = 0.046), with values in the AP direction significantly higher when viewing images passively than when viewing them actively in the same direction. Lastly, a significant three-way interaction effect was found between stimulation, valence, and direction (F(1, 35) = 7.406, *p* = 0.010) (Table 2).

## 4. Discussion

For this study, we had the hypotheses of a differential modulation of non-linear measures of COP’s displacement both with the stimulus valence (painful vs. non-painful) and vision condition (active vs. passive) factors. To the best of our knowledge, this study is the first to report the non-linear modulation of postural control within the context of observed/imagined pain. These findings complement previous studies that have demonstrated the differential modulation of postural control in response to observed/imagined pain using linear analysis methods [5,6,7]. It is important to note that non-linear methods provide insight into the dynamics of this modulation of postural control rather than its specific nature.

Our results indicate a significant effect of valence (painful vs. non-painful) on LyE but not on SampEn, mostly in the anteroposterior (AP) direction, with a significant interaction between the stimulation and valence factors. This suggests a specific influence of valence on the dynamics of the chaotic behavior of postural control. In response to the embodiment of painful stimuli, individuals may be more inclined to stiffen their posture, rendering them less sensitive to potential environmental disturbances [10].

Among the non-linear indices, our analyses indicate a difference between SampEn, which appeared to be irrelevant for discriminating the modulation of postural control during the perception of painful stimuli, and FD and LyE, which both revealed significant differences between painful and non-painful conditions. Regarding the direction of postural control, the analyses revealed the presence of significant differences for FD and LyE in the AP, as compared to the ML direction. These results are in accordance with previous findings [10,13] and highlight the pertinence and usefulness of posturography to assess approach–avoidance-type behaviors in motivational and emotional processes.

Although both the AP and ML directions can be considered to explore posturographic control modulation, the AP direction seems to be a particularly useful biomarker for examining modulation within the framework of socio-affective information processing. In the AP position, we observed a significant effect of the stimulation factor (passive viewing vs. incarnation) on LyE and FD (quasi-significant for SampEn), with higher values observed in the passive conditions compared to the active ones. These findings support the role of incarnation and embodiment in modulating postural control induced by observed/imagined pain [6], as well as the specific influence of this cognitive process on the temporal course of this modulation (sometimes leading to a reversal of approach–avoidance behavior). According to these results, actively incorporating visual scenes could contribute to less optimal postural control (lower FD) and the development of rigidity, translating into less flexible postural adaptation and an inability to promptly adjust to new environmental changes. This freezing strategy was hypothesized to explain the prioritization of bodily responses during the interaction between the perception of a postural threat and an aversive visual stimulus [21].

First, we proposed to interpret this result as an increased self-protective strategy in response to painful stimuli. However, it is quite obvious that passive and active stimulation conditions do not mobilize the same cognitive resources, with attentional resources disengaged from postural control in the active condition [22]. Essentially, cognitive resources, including attentional ones, would be distributed to both postural control and stimulus processing, and giving an instruction to imagine oneself in the scene would mobilize a portion of these resources that would no longer be available for postural control. In previous studies, the active condition was introduced under the assumption that it would potentiate the embodiment of the stimuli, which was assumed to be necessary in the development of postural correlates of socio-affective stimuli processing. Our results raise the question of how automatic and early the embodiment of stimuli is and whether it is necessary for a coherent postural response to painful stimuli. This question cannot be answered based on the current results. In future studies, it would be necessary to compare experimental conditions in which cognitive resources (and in particular attentional resources) involved in postural control are mobilized in identical ways. It would also be very interesting to incorporate objective measures, such as questionnaires or biological markers, of the embodiment process, which we assume to be important in postural control in relation to emotions and motivations.

It is important to note several limitations of this study. Firstly, although non-linear measurements provide information on the dynamics of the postural response, they do not provide any information on the nature of the postural reactions (approach–avoidance) or the exact timing of the response. Secondly, the study did not measure any central activity during postural modulation within the different experimental conditions. It would have been particularly interesting to have information on the neural processes involved in the dynamics of postural control.

In conclusion, our results are in accordance with (i) a differential modulation of postural control in response to painful stimuli as compared to non-painful ones; (ii) an influence of cognitive resources on this modulation (further studies will be necessary to clarify the role of embodiment in this modulation); (iii) an emphasis on LyE among other non-linear measures for assessing postural modulation in response to painful stimuli perception.

## 5. Conclusions

This study is the first to demonstrate the utility of non-linear measures, especially LyE, in investigating the postural correlates of painful stimulus perception and the role of the embodiment process. Our results underscore the importance of examining the temporal aspects of postural responses in the field of affective neuroscience.

Once we have reached these conclusions, it becomes important to consider how the findings can be put into practice. One promising application is in the field of clinical medicine. Several studies have demonstrated the usefulness of using non-linear measures of postural control, including (i) for patients with multiple sclerosis [23], (ii) after total hip or knee arthroplasty [24], and (iii) for certain syndromes [25]. It is worth noting that many neurological diseases first manifest as the impairment of emotional, motivational and social functions, before cognitive functions are affected. Therefore, it is essential to identify biomarkers that can be easily measured in clinical settings, which can help to diagnose the impairment of these functions in patients. By quickly calculating non-linear measures related to patients’ postural control in response to non-painful versus painful images (especially the Lyapunov coefficient), it may be possible to identify such a biomarker.

## Figures and Tables

**Table 1:** Interaction effect of stimulation and direction on FD values.

Relationship	Values for Relationship	*p*-Value
Passive, AP > Active, AP	2.531 ± 0.144 > 2.514 ± 0.170	<0.001
Passive, AP > Passive, ML	2.531 ± 0.144 > 2.555 ± 0.145	<0.001
Passive, AP > Active, ML	2.531 ± 0.144 > 2.459 ± 0.166	<0.001
Active, AP > Active, ML	2.514 ± 0.170 > 2.459 ± 0.166	<0.007

**Table 2:** Interaction effect of stimulation, valence and direction on LyE values.

Relationship	Values for Relationship	*p*-Value
Passive, painful, AP > Active, non-painful, AP	2.531 ± 0.144 > 2.418 ± 0.100	0.008
Active, painful, AP > Active, non-painful, AP	2.513 ± 0.117 > 2.418 ± 0.100	0.003
Passive, non-painful, AP > Active, non-painful, AP	2.514 ± 0.170 > 2.418 ± 0.100	0.029
Active, non-painful, AP < Passive, painful, ML	2.418 ± 0.100 < 2.555 ± 0.145	<0.001
Active, non-painful, AP < Active, painful, ML	2.418 ± 0.100 < 2.548 ± 0.128	0.002
Passive, painful, ML > Passive, non-painful, ML	2.555 ± 0.145 > 2.459 ± 0.166	0.002

## Data Availability

The data presented in this study are available on request from the corresponding author.
